# Effect of n-3 Polyunsaturated Fatty Acids Enriched Chicken Meat Consumption in Relation to Oxidative Stress Marker Levels in Young Healthy Individuals: A Randomized Double-Blind Study

**DOI:** 10.3390/antiox14020204

**Published:** 2025-02-11

**Authors:** Tihana Nađ, Nikolina Kolobarić, Zrinka Mihaljević, Ines Drenjančević, Petar Šušnjara, Ana Stupin, Darjan Kardum, Zlata Kralik, Gordana Kralik, Manuela Košević, Ivana Jukić

**Affiliations:** 1 Clinic of Pediatrics, University Hospital Centre Osijek, J. Huttlera 4, HR-31000 Osijek, Croatia; tnad@mefos.hr; 2 Department of Pediatrics, Faculty of Medicine Osijek, Josip Juraj Strossmayer University of Osijek, J. Huttlera 4, HR-31000 Osijek, Croatia; darjan.kardum@mefos.hr; 3 Department of Physiology and Immunology, Faculty of Medicine Osijek, Josip Juraj Strossmayer University of Osijek, J. Huttlera 4, HR-31000 Osijek, Croatia; nbdujmusic@mefos.hr (N.K.); zmihaljevic@mefos.hr (Z.M.); idrenjancevic@mefos.hr (I.D.); ana.stupin@mefos.hr (A.S.); 4 Scientific Centre of Excellence for Personalized Health Care, University of Osijek, Trg Sv. Trojstva 3, HR-31000 Osijek, Croatia; zlata.kralik@fazos.hr (Z.K.); gkralik@fazos.hr (G.K.); manuela.kosevic@fazos.hr (M.K.); 5 Faculty of Kinesiology, Josip Juraj Strossmayer University of Osijek, Drinska 16a, HR-31000 Osijek, Croatia; psusnjara@kifos.hr; 6 Department of Neonatology, Zadar General Hospital, B. Peričića 5, HR-23000 Zadar, Croatia; 7 Department of Animal Production and Biotechnology, Faculty of Agrobiotechnical Sciences, Josip Juraj Strossmayer University of Osijek, Vladimira Preloga 1, HR-31000 Osijek, Croatia

**Keywords:** functional food, fatty acids, dietary intake, reactive oxygen species, antioxidant defense

## Abstract

Oxidative stress and inflammation are considered important risk contributors for various diseases. Over the last few decades, increasing attention has been focused on the role of n-3 polyunsaturated fatty acids (n-3 PUFAs) in human health and disease. We aimed to evaluate the effect of n-3 PUFA-enriched chicken meat consumption (~1500 mg of n-3 PUFAs intake per day) for three weeks on oxidative status and antioxidative capacity in young healthy individuals. This was a randomized, double-blinded, controlled trial, in which thirty-nine young healthy people were randomly allocated to eating 500 g/day of regular chicken meat (Control group) or n-3 PUFA-enriched chicken meat (n-3 PUFAs group) over 3 weeks. Subjects’ biochemical parameters, including serum lipids level, liver enzymes, serum activities of antioxidant enzymes (glutathione peroxidase (GPx), superoxide dismutase (SOD)), serum oxidative stress markers (thiobarbituric acid reactive substances (TBARS) and ferric-reducing ability (FRAP)), as well as intracellular production of reactive oxygen species (ROS) in peripheral blood mononuclear cells, were assessed before and after completing the three-week dietary protocol. N-3-enriched chicken meat consumption significantly reduced high-sensitivity C reactive protein (hsCRP) serum level and increased the level of the antioxidant defense marker, FRAP. Furthermore, GPx and SOD enzyme activities significantly increased in the n-3 PUFAs group compared to baseline, which was accompanied by significantly decreased ROS production. In healthy young individuals, the 3-week dietary intake of n-3 PUFA-enriched chicken meat significantly increased the serum total antioxidant and anti-inflammatory potential, indicating that n-3 PUFAs may be protective in resting health condition without inflammatory processes.

## 1. Introduction

Functional food represents one of the most intensively studied and widely promoted areas in food and nutritional science. While functional food is known as a food offering potential health benefits or disease prevention alongside its nutritional value [1], it is essential to understand that it cannot compensate for poor lifestyle habits. Functional food is considered one of the aspects of an extensive approach to achieving good health [2].

The long-chain n-3 polyunsaturated fatty acids (n-3 PUFAs), specifically docosahexaenoic acid (DHA) and eicosapentaenoic acid (EPA), are nutrients necessary for numerous metabolic and physiological human processes that have many positive effects on human health [3,4]. The major sources of n-3 PUFAs in the daily diet are marine organisms (particularly fatty fish) but also some vegetables, nuts, and seeds (walnuts, sunflower, chia seeds, etc.) [5,6,7]. In addition, a significant source of n-3 PUFAs available on the market is various n-3 PUFA rich dietary supplements and functional foods (chicken eggs, enriched pasta, cereals, etc.) [8].

Studies have demonstrated that n-3 PUFAs have a major role in altering serum lipid levels and influence eicosanoid synthesis, cell signaling, and gene expression, thereby affecting health [4,9]. The favorable effect of n-3 PUFAs has also been investigated in patients with various diseases, such as cardiovascular (CV) diseases (including atrial fibrillation, atherosclerosis, and thrombosis), autoimmune diseases, and mental disorders [4,10,11]. Furthermore, the effects of long-chain n-3 fatty acid supplementation in pregnancy, lactation, and infancy have been thoroughly studied [12,13,14]. Although the evidence for the beneficial effect of n-3 PUFAs on some disorders (including depression and cancer) is not conclusive [15,16], evaluation remains difficult due to the heterogeneity of the populations studied and the interventions used. The recommendations for daily n-3 PUFA intake depend on age and the physiological and health status [17], and, therefore, the recommended values differ for the general (healthy) population and those with some (patho)physiological conditions. Still, based on all the evidence, the US Food and Drug Administration (FDA) recommends no more than 3 g/day of EPA and DHA combined, with up to 2 g/day from dietary supplements [18].

While the mechanisms behind the beneficial effects of n-3 PUFAs on human health are not fully elucidated and are still being intensively researched, their antioxidant and anti-inflammatory properties are suggested to be the most important preventive and therapeutic effects [6,19]. The most notable features of n-3 PUFA’s antioxidant and anti-inflammatory activity are competition with arachidonic acid for enzymes involved in the biosynthesis of pro-inflammatory mediators [20], inhibition of pro-inflammatory nuclear factor kappa B (NF-κ B) activation [21], and metabolism to pro-resolution lipid mediators [22,23]. Resolvins (Rv) are small molecules formed from n-3 fatty acids that can promote the resolution of inflammation since they decrease pro-inflammatory gene expression and mediators [24]. Furthermore, they can prevent the accumulation of neutrophils at the site of inflammation and improve the elimination of pathogens and apoptotic cells, thereby preventing the development of excessive inflammation and accelerating the recovery of tissue homeostasis [25,26]. The Rv-D series derives from DHA and/or n-3 docosapentaenoic acid, while the Rv-E series derives from EPA [27]. Available data indicate that the ability of EPA and DHA to produce primarily pro-resolving lipid mediators can be of crucial importance for their well-known health benefits [24].

Oxidative stress represents a (patho)physiological condition defined as a disturbed balance between the reactive oxygen species (ROS) production and the organism’s ability to detoxify them. Increased oxidative stress is harmful and has been implicated in the pathogenesis of numerous diseases including CV disease [28]. Therefore, serum circulating biomarkers of inflammation and oxidative stress are considered risk indicators for the development of these diseases. An antioxidant-rich diet has been demonstrated to provide protective actions by scavenging free radicals and increasing the activity of antioxidant enzymes, leading to improved clinical outcomes [29,30]. Though there are some contradictory reports concerning the effects of PUFA supplementation on oxidative stress levels, it is suggested that n-3 PUFA may increase total antioxidative capacity and reduce oxidative injury [31,32,33]. Furthermore, our research group has also previously demonstrated that supplementation with n-3 PUFAs may affect physiological processes related to oxidative balance. Dietary consumption of nutritionally n-3 PUFA-enriched hen eggs decreased oxidative stress and also modified immune response to favor inflammation resolution [34,35,36].

According to the available prospective observational studies and randomized clinical trials, the favorable antioxidant/anti-inflammatory effects of n-3 PUFA supplementation seem to be most consistent for CV disease [37], while the potential effects in a general healthy organism without any diseases or inflammatory processes are more or less well established. Furthermore, previous studies have examined the effect of n-3 PUFA supplementation, mainly in capsule form [38,39], while data on the effect of functional food enriched with n-3 PUFAs are lacking in young and healthy people. Based on our previous research, in which we reported the beneficial effect of n-3 PUFA-enriched hen eggs in healthy individuals and also found that consumption of three regular eggs/day is safe for healthy populations [36], the focus of this study was to evaluate the potential beneficial effect of n-3 PUFAs applied in the form of enriched chicken meat. Thus, the main objective of this study was to investigate whether there is a beneficial effect of the consumption of n-3 PUFAs, in the form of enriched chicken meat, on the body’s oxidative status in young and healthy people, without any disease or inflammatory condition. More specifically, we aimed to estimate if n-3 PUFA-enriched functional food consumption affected the resolvins metabolism, activity of antioxidant enzymes, or intracellular ROS production.

## 2. Materials and Methods

### 2.1. Study Protocol and Interventions

This randomized, double-blind, placebo-controlled study (registered at clinicaltrials.gov, NCT05725486) was carried out at the Department of Physiology and Immunology, Faculty of Medicine in Osijek, Croatia.

**Figure 1:**
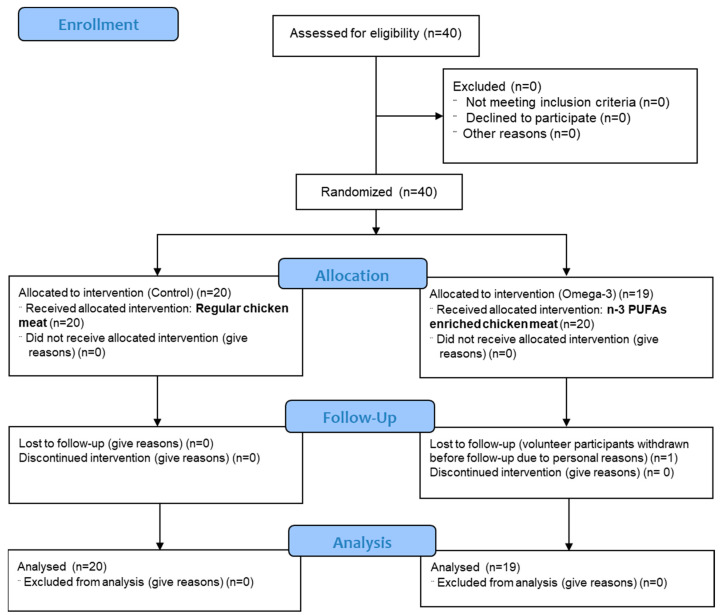
CONSORT 2010 flow diagram.

Forty healthy young people of both sexes, aged between 20 and 26 years, were assessed as eligible for participation, one of which did not complete the protocol for personal reasons. Finally, 39 healthy young volunteers participated in this study (Figure 1). Exclusion criteria were diabetes, hypertension, hyperlipidemia, cerebrovascular or renal diseases, coronary or peripheral artery disease, chronic inflammatory disorders, history of smoking, and use of medications that might influence the endothelium. None of the subjects consumed any (not only n-3 PUFAs) supplements or functional food before or during the experiment.

The study protocol and procedures conformed to the latest revision of the Declaration of Helsinki and were approved by the Ethical Committee of the Faculty of Medicine, University of Osijek (CLASS: 602-04/23-08/03; Reg. No.: 2158-61-46-23-125). The study protocol was explained in detail to all subjects, and they had to sign informed consent before inclusion in this study. All participants were explicit volunteers, and no compensation was provided for their participation in the study. This study followed the CONSORT guidelines for randomized controlled trials. Details regarding adherence can be found in the Supplementary Materials (Table S1).

The study protocols lasted for three weeks, during which all subjects daily ate 500 g of chicken meat. Subjects were randomly assigned (randomization was completed using a coin (letter-1 or head-2) by an unbiased person who was not involved in the performed analyses) to one of the two study groups: control group (n = 20; W/M = 12/8) consumed regular chicken meat (breast and thigh muscle, n-3 PUFAs content ~118 mg/day), whereas n-3 PUFAs group (n = 19; W/M = 8/11) ate n-3 PUFA-enriched chicken meat (breast and thigh muscle, n-3 PUFAs content ~1500 mg/day). The participants had two study visits, during which all measurements were taken: the first visit was on the first day of the study (before starting consumption of the chicken meat) and the second visit was on the day immediately following the end of the protocol. All participants kept a diet diary and did not eat any other or additional meat throughout the study protocol.

The person who was involved in randomization sent the necessary information to an unbiased associate who did not have personal data but previously assigned labels: 1—control group that ate regular chicken meat; 2—n-3 PUFAs group that ate enriched chicken meat with n-3 PUFAs. Therefore, the chicken meat was indistinguishable; both participants and investigators were blinded throughout the study.

The enriched chicken meat was produced and provided by the Faculty of Agrobiotechnical Sciences, University of Osijek, by substitution of sunflower oil (5%) with a mixture of fish (1%), linseed (3%), and rape (1%) oil in feed mixtures. To determine the profile of fatty acids, meat samples were prepared with a MARS 6 microwave device (CEM Corporation, Matthews, NC, USA) using microwave power of 1200 W and high temperature. After the reaction, the samples were cooled and extracted in pentane, transferred to a vial and stored in the freezer until analysis.

Chromatographic analysis was performed on a SCION 436-GC gas chromatograph (SCION Instruments, Goes, The Netherlands) equipped with a flame ionization detector (FID). A FAMEWAX (Restek Corporation, Bellefonte, PA, USA) capillary column (30 m × 0.32 mm ID × 0.25 µm df) was used for the separation of fatty acids. The injection volume was 1 µL, and the operating conditions were as follows: injector temperature: 230 °C, detector temperature 230 °C, carrier gas flow (hydrogen) 2.5 mL/min. The oven temperature program was programmed as follows: from 50 to 160 °C 20 °C/min, from 160 to 225 °C 10 °C/min with a hold at 225 °C for 9 min. The total analysis time was 21 min. A standard mixture of 37 fatty acids (Food Industry FAME Mix, Restek Corporation, Bellefonte, PA, USA) was used to identify individual fatty acids in the chromatogram. The proportions of individual and total fatty acids are presented as a percentage of total fatty acids in meat lipids.

On the first day of this study, after taking the initial measurements, the participants received all the meat needed for the study protocol, which was prepackaged. Each package contained a precise daily amount of meat; 400 g of chicken breast and 100 g of chicken thigh muscle. Participants were instructed to eat one package of meat every day, prepared as boiled or short-seared on both sides in a drop of olive oil. They were also prohibited from taking additional meat or n-3 PUFA supplementation in any form before enrolment or during the study protocol.

### 2.2. Anthropometric Measurements and Venous Blood Laboratory Analysis

To calculate the waist-to-hip (WHR) ratio and body mass index (BMI), all participants’ height, weight, and hip and waist circumferences were measured. At both study visits, a venous blood sample was taken from each participant, and using standardized laboratory protocols, their full blood count, plasma electrolytes (potassium, sodium, calcium), creatinine, urea, iron, transferrin, high-sensitivity C reactive protein (hsCRP), fasting blood glucose, liver (aspartate aminotransferase (AST), alanine aminotransferase (ALT) and gamma-glutamyl transferase (GGT)) and lipid biomarkers (triglycerides, low-density lipoprotein cholesterol (LDL), and high-density lipoprotein cholesterol (HDL) and total cholesterol) were determined.

### 2.3. Serum Isolation and PBMC Processing: Isolation, Cryopreservation, and Thawing

The protocol is described in detail in our previous papers [34,40]. Briefly, for serum isolation, blood samples were obtained in a 6 mL vacutainer clot activator tube (BD Vacutainer, Becton, Dickinson and Company, Franklin Lakes, NJ, USA), centrifuged, and aliquoted in an appropriate number of microcentrifuge tubes. Aliquots were stored in designated boxes at −80 °C before other proceedings.

Peripheral blood mononuclear cells (PBMCs) were isolated from venous blood samples collected in EDTA-containing vacutainer tubes (BD Vacutainer, Becton, Dickinson and Company, Franklin Lakes, NJ, USA). PBMCs were isolated within three hours, with reagents warmed to RT (20–25 °C) before use. Then, 16 mL of blood was diluted 1:1 with PBS, layered on Ficoll-Paque^®^ PLUS (GE Healthcare Bio-Sciences AB, Uppsala, Sweden, Lot: 1029693), and centrifuged (800 G/25 min with brakes off). PBMCs, visible as a cloudy ring, were collected, rinsed in PBS, and their viability was assessed using Trypan blue (Sigma-Aldrich, Merck KGaA, Darmstadt, Germany, Lot: RNBH9907) staining and a Bürker-Türk chamber.

For cryopreservation, a 1:9 ratio of dimethyl sulfoxide (DMSO; Supelco, Merck KGaA, Darmstadt, Germany) and fetal bovine serum (FBS; Sigma-Aldrich, St. Louis, MA, USA) was used. Cryovials with samples were placed in a Mr. Frosty freezing container (Nalgene Labware, Thermo Fisher Scientific, Waltham, MA, USA) filled with isopropyl alcohol and stored at –80 °C (24 h) before transferring them to labeled storage containers. Thawing was performed using RPMI-1640 culture medium (Capricorn Scientific GmbH, Ebsdorfergrund, Germany), supplemented with FBS and penicillin-streptomycin antibiotic (1%) (Capricorn Scientific GmbH, Ebsdorfergrund, Germany), preheated to ~37 °C.

### 2.4. Evaluation of Biomarkers of Oxidative Stress and Antioxidative Defense

According to the previously described protocol [41,42], serum biomarkers of oxidative stress were determined using spectrophotometry. Lipid peroxidation products are indicators of the level of created oxidative stress, which are measured using TBARS (thiobarbituric acid reactive substances) method, whereas the ferric-reducing ability of plasma (FRAP) method is considered a serum biomarker of the blood’s antioxidant capacity. Briefly, venous blood samples were collected during both study visits in tubes containing anticoagulants, frozen in liquid nitrogen, and stored in a refrigerator at -80 °C until the experiments were performed. For the TBARS measurements, the sample was mixed with trichloroacetic acid to precipitate the proteins. For further determinations, supernatant was used. Nanophotometer P300 UV/VIS, IMPLEN at 572 and 532 nm was utilized, and, using the malondialdehyde (MDA) as a standard (µM MDA) [43], the absorbance of the sample was measured. In the FRAP method, Fe^3+^-TPTZ (2,4,6-tris(2-pyridyl)- s-triazine) was reduced to Fe^2+^-TPTZ in the presence of antioxidants, and a blue discoloration occurred. The sample’s absorbance was determined with the Nanophotometer P300 UV/VIS, IMPLEN at 593 nm as standard (mM/L Trolox) [44].

### 2.5. Analysis of Serum Antioxidant Enzyme Activities

Enzyme activity assay in undiluted serum samples was performed using a Spark multimode microplate reader with SparkControl software version 2.1 (Tecan, Männedorf, Switzerland) according to the established protocol with adjusted volumes and extinction factor for microplate reader at the Subdepartment for Biochemistry and Molecular biology; Department of Biology, J. J. Strossmayer University of Osijek [45]. Total superoxide dismutase (SOD) activity is measured as the degree of inhibition of cytochrome C reduction by a superoxide radical. One unit of SOD inhibits the cytochrome C reduction rate by 50%, in a linked system using xanthine oxidase (XOD) and xanthine. The superoxide radical, formed enzymatically in a reaction catalyzed by XOD, is reduced by cytochrome C, and the reduction rate is monitored at 550 nm. SOD inhibits cytochrome C reduction by dismuting the superoxide radical. SOD activity assay was performed according to the modified method first described by Flohe and Otting [46]. The serum activity of glutathione peroxidase (GPx) was determined indirectly by measuring the rate of oxidation of nicotinamide adenine dinucleotide phosphate (NADPH) to NADP+, accompanied by a decrease in absorbance at 340 nm for 5 min. One unit of GPx activity catalyzes the oxidation of 1.0 μmol of reduced glutathione by H_2_O_2_ to oxidized glutathione per minute at pH of 7.0 and 25 °C according to Wendel’s protocol [47].

The measured enzyme’s activities were presented as units of enzymes per milligram of protein (U/mg protein). The serum protein concentration (mg/mL) was determined at 595 nm according to the manufacturer protocol for Bradford reagent (Bradford Reagent B6916, Sigma Aldrich), using bovine serum albumin as a standard.

### 2.6. Intracellular Reactive Oxygen Species (ROS) Production Measurements

Intracellular ROS production in PBMCs was assessed using a FACS Canto II flow cytometer (BD Bioscience) and Flow Logic software (v8, Inivai Technologies, Mentone, Australia) following standard protocols [42,48,49]. DCF-DA-mediated fluorescence measured hydrogen peroxide (H_2_O_2_) and peroxynitrite (ONOO–), while DHE-mediated fluorescence detected superoxide (O_2_^−^) in PBMCs. ROS production was further stimulated by adding phorbol 12-myristate 13-acetat (PMA). PBMC samples (1 × 10^6^ cells) were rinsed, resuspended in PBS, and stained (final concentration 10 µM), followed by a 30 min incubation at +4 °C in the dark. After incubation, DCF-DA samples were resuspended in PBS and analyzed via flow cytometry, while DHE samples underwent an additional rinsing step before being resuspended in PBS for analysis.

### 2.7. ELISA Assay

Serum concentrations of resolvin E1 (RvE1) (Cat: MBS744335; MyBioSource Inc., San Diego, CA, USA; sensitivity: 1.0 pg/mL) and resolvin D1 (RvD1) (Cat: MBS756429; MyBioSource Inc., CA, USA; sensitivity: 1.0 pg/mL) were determined using commercially available enzyme-linked immunosorbent assay (ELISA) kits on compact absorbance reader for 96-well microplates (BioRad PR 3100 TSC, Bio-Rad Laboratories, Hercules, CA, USA).

### 2.8. Statistical Analysis

All results were reported as mean and standard deviation (SD). To assess the normality of the data distribution, the Kolmogorov–Smirnov normality test was used. For assessing the differences within the groups (measurements before and after study protocol), the Wilcoxon rank-sum test was used in the case when variables were not normally distributed, while the paired *t*-test was used for normally distributed data. Differences between groups in measurement at the end of the protocol were tested using analysis of covariance (ANCOVA) with adjustments for baseline (premeasurement). Fatty acid profile of chicken meat (regular and n-3 PUFA-enriched) was analyzed by one-way analysis of variance (ANOVA). *p* < 0.05 was considered statistically significant. For statistical analysis, SigmaPlot version 15 (Systat Software, Inc., Chicago, IL, USA) was used. All graphs were made using GraphPad Prism6 (GrafPad Software, Inc., San Diego, CA, USA).

## 3. Results

### 3.1. Fatty Acid Profile of Chicken Meat

The composition of fatty acids in chicken breast and chicken thigh muscle of regular (consumed by controls) and n-3 PUFA-enriched meat (consumed by n-3 PUFAs group) is shown in Table 1. By changing the proportion of fatty acids in the feeding mixture, the concentration of n-6 was reduced, and the concentration of n-3 PUFAs was increased in chicken meat (Table 1).

### 3.2. General Characteristics and Biochemical Measurements of Participants

There were no differences between study groups regarding their daily dietary habits; the total intake of carbohydrates, proteins, and fats, as well as their main sources, was similar in both groups. The only difference was in the type of chicken meat consumed. Table 2 summarizes the general characteristics and biochemical measurements of the participants. There was no sex difference between the examined groups (*p* = 0.527); in the control group, there were 12 women and 8 men, whereas in the n-3 PUFA group, there were 8 women and 11 men. Both groups were age-matched (23 ± 3 years old). All participants were lean and had normal full blood counts, liver or renal function, serum electrolytes, fasting lipid and glucose levels, and hsCRP. The basal values of all measured parameters (age, BMI, WHR, and biochemical parameters) did not differ between the examined groups.

The effects of regular or n-3 PUFA-enriched chicken meat consumption on anthropometric characteristics and biochemical measurements are shown in Table 2. Analysis of all measured parameters showed that, although the basal values were also within the reference range, leukocytes and hsCRP significantly decreased after consumption of n-3 PUFA-enriched chicken meat, while there was no influence of meat consumption (regular or n-3 PUFA-enriched) on other measured parameters compared with baseline measurements, anthropometric (BMI and WHR) or biochemical (plasma electrolytes, kidney and liver parameters, fasting blood glucose, serum lipid profiles). Furthermore, these measurements did not differ between groups.

### 3.3. Serum Level of Oxidative Stress and Antioxidative Defense Biomarkers

**Figure 2:**
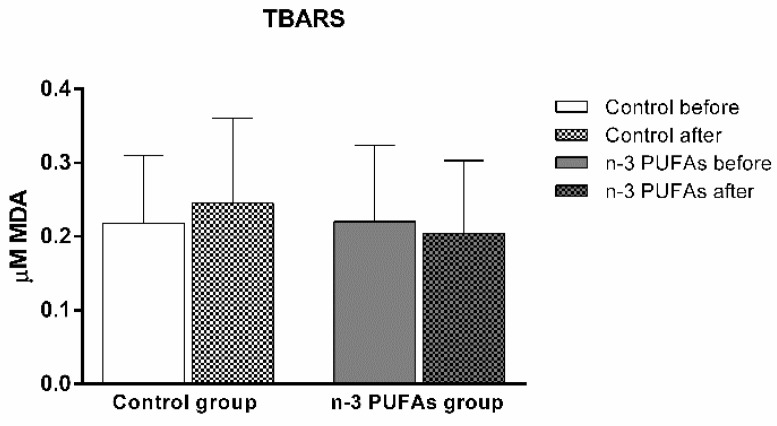
The impact of a three-week dietary intake of regular (control group) and n-3 PUFA-enriched (n-3 PUFAs group) chicken meat on serum levels of lipid peroxidation marker TBARS. Data are expressed as mean and standard deviation (SD). Paired *t*-test; analysis of covariance (ANCOVA).

The serum levels of the lipid peroxidation marker TBARS were not significantly changed with either regular or n-3 PUFA-enriched chicken meat consumption compared to baseline. Additionally, TBARS plasma levels were similar between the groups (Figure 2).

**Figure 3:**
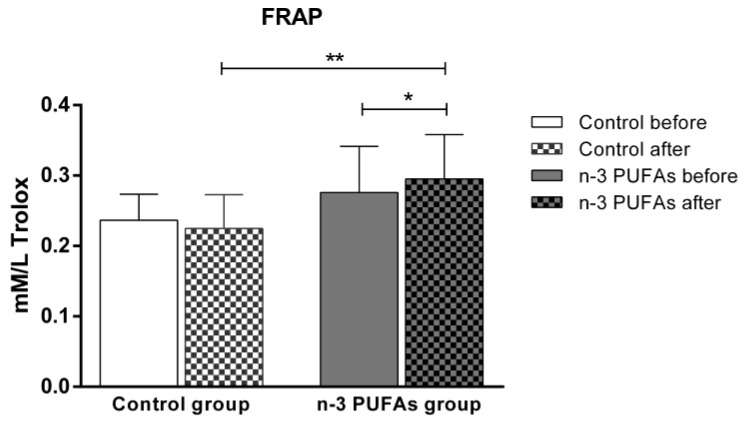
The impact of a three-week dietary intake of regular (control group) and n-3 PUFA-enriched (n-3 PUFAs group) chicken meat on serum levels of antioxidative defense biomarker FRAP. Data are expressed as mean and standard deviation (SD). * *p* < 0.05 before vs. after within the n-3 PUFAs group—paired *t*-test; ** *p* < 0.05 control vs. n-3 PUFAs group—analysis of covariance (ANCOVA).

A 21-day dietary intake of chicken meat enriched with n-3 PUFAs significantly increased the FRAP level (a marker of antioxidant defense) compared to the baseline and to the control group (Figure 3). In contrast, the FRAP levels remained unchanged in the control group after consuming regular chicken meat throughout the study protocol.

### 3.4. Antioxidant Enzyme Activity in Serum Samples

The consumption of n-3 PUFA-enriched chicken meat for 21 days significantly increased the serum activities of GPx and SOD compared to baseline (Table 3), while regular chicken meat consumption did not have a significant effect on enzyme activities in the control group.

### 3.5. Assessment of Intracellular Reactive Oxygen Species Production

**Figure 4:**
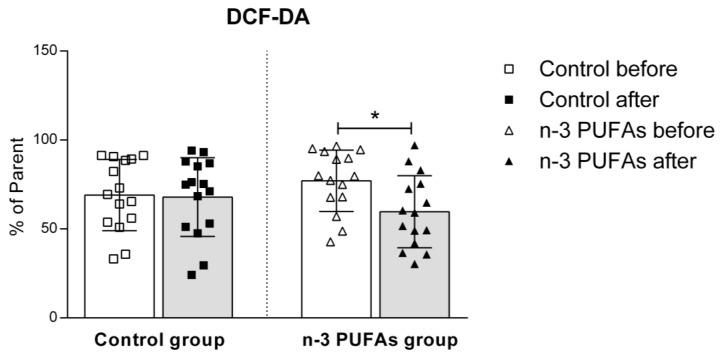
The effect of regular (control) and n-3 PUFA-enriched (n-3 PUFAs) chicken meat dietary intake on intracellular production of hydrogen peroxide and peroxynitrite (DCF-DA) in peripheral blood mononuclear cells (PBMCs) in young healthy participants. Data are presented as mean and standard deviation (SD). * *p* < 0.05 before vs. after within the n-3 PUFAs group; paired *t*-test.

**Figure 5:**
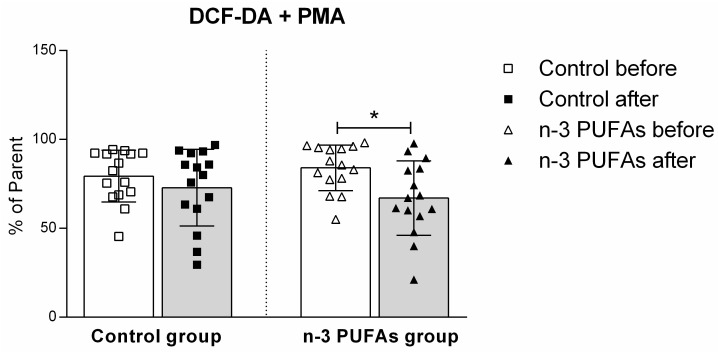
The impact of regular (control) and n-3 PUFA-enriched (n-3 PUFAs) chicken meat consumption on intracellular production of hydrogen peroxide and peroxynitrite (DCF-DA) in peripheral blood mononuclear cells (PBMCs) in young healthy participants, following PMA stimulation. Data are presented as mean and standard deviation (SD). * *p* < 0.05 before vs. after within the n-3 PUFAs group; paired *t*-test.

Hydrogen peroxide and peroxynitrite production in PBMCs significantly decreased in the n-3 PUFAs group following dietary treatment, both with and without PMA stimulation (Figure 4 and Figure 5). Regular chicken meat dietary intake did not have any significant effects on the production of hydrogen peroxide or peroxynitrite in PBMCs.

**Figure 6:**
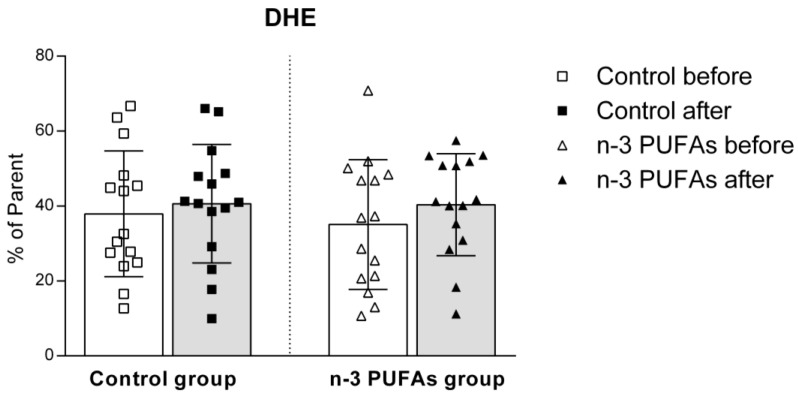
The impact of regular (control) and n-3 PUFA-enriched (n-3 PUFAs) chicken meat consumption on intracellular production of superoxide (DHE) in peripheral blood mononuclear cells (PBMCs) in young healthy participants. Data are expressed as mean and standard deviation (SD). paired *t*-test; analysis of covariance (ANCOVA).

**Figure 7:**
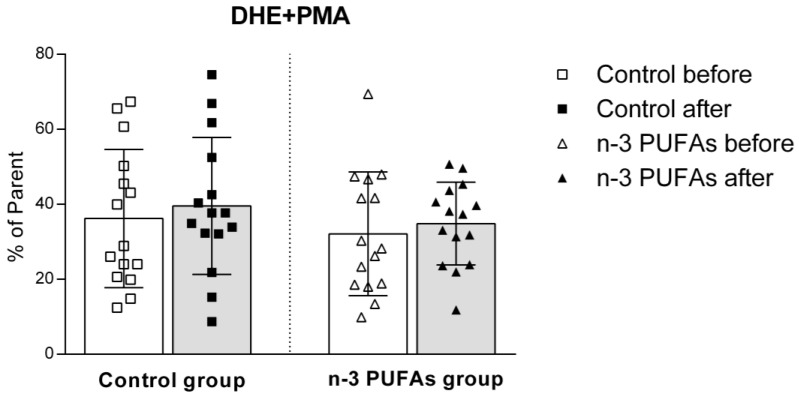
The effect of regular (control) and n-3 PUFA-enriched (n-3 PUFAs) chicken meat dietary intake on intracellular production of superoxide (DHE) in peripheral blood mononuclear cells (PBMCs) in young healthy participants, following PMA stimulation. Data are expressed as mean and standard deviation (SD). Paired *t*-test; analysis of covariance (ANCOVA).

The dietary intervention had no impact on superoxide production in either the control or n-3 PUFAs groups, regardless of PMA stimulation (Figure 6 and Figure 7).

### 3.6. Serum Concentrations of RvE1 and RvD1

**Figure 8:**
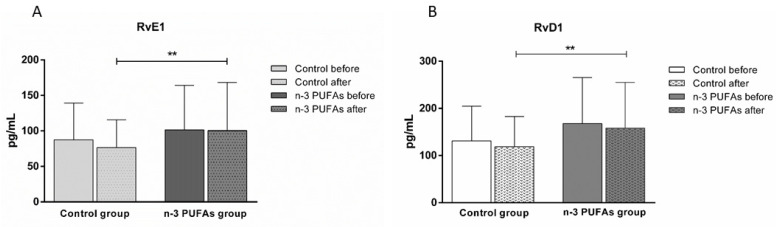
The impact of regular (control group) and n-3 PUFA-enriched (n-3 PUFAs group) chicken meat dietary intake on serum levels of serum RvE1 (panel **A**) and RvD1 (panel **B**) concentrations. Data are expressed as mean and standard deviation (SD). ** *p* < 0.05 control vs. n-3 PUFAs group—analysis of covariance (ANCOVA).

A three-week dietary protocol of n-3 PUFA-enriched chicken meat consumption significantly increased both RvE1 and RvD1 serum concentrations compared to the values of the control group, obtained after the consumption of regular chicken meat (Figure 8).

## 4. Discussion

To the best of our knowledge, this was the first randomized double-blinded placebo-controlled interventional study investigating the impact of n-3 PUFA-enriched chicken meat consumption on oxidative status and antioxidant potential in healthy young individuals without any accompanying comorbidities. The salient findings of this study are that in healthy young people, the daily consumption of n-3 PUFA-enriched chicken meat for three weeks (a) significantly reduced hsCRP levels; (b) significantly enhanced antioxidant defense; (c) significantly increased antioxidant enzyme activities; (d) significantly reduced the production of hydrogen peroxide and peroxynitrite in PBMCs; and (e) significantly enhanced serum production of resolvins, RvE1 and RvD1. On the other hand, the consumption of standard regular chicken meat for 21 days showed no changes in the measured parameters.

In our earlier study involving healthy young individuals who ate three eggs daily for 3 weeks, we reported that n-3 PUFA-enriched chicken eggs (approximately 777 mg of n-3 PUFAs/day) were accompanied by decreased serum triglycerides and hsCRP levels [50]. In the present study, in which young healthy participants consumed n-3 PUFAs in the form of enriched chicken meat, the hsCRP level was also significantly decreased (Table 2), whereas we found no significant difference in the serum lipid levels (including triglycerides). It is important to point out that all study volunteers were young and healthy people who showed basal lipid values that were within the normal standard range. However, the other authors present inconsistent results on the impact of n-3 PUFAs on lipid levels in healthy people [51,52,53], while in patients with increased CV risk, most of the studies suggest that supplementation with n-3 PUFAs may be a useful dietary approach for improving lipid profiles [51,54]. To our knowledge, there are only a few studies investigating the effect of n-3 PUFA-enriched chicken meat on health status in young healthy people [55,56,57], but only one study investigated the effect of enriched chicken meat on serum lipid levels in healthy volunteers [56]. The results showed that a two-month daily consumption of n-3 fatty acid-enriched eggs and chicken meat for eight weeks does not affect the elementary body parameters, such as body weight, and also the plasma lipids (including cholesterol, triglycerides, HDL, and LDL), which is consistent with the results of this study. Although it is accepted that n-3 PUFAs reduce the plasma lipid levels in humans [58,59] by inhibiting crucial enzymes involved in hepatic triglycerides biosynthesis [60], the n-3 PUFA effects certainly depend on the forms, dose, and duration of supplementation.

Circulating inflammatory mediators, including hsCRP, are linked to a higher risk of CV events, independent of cholesterol and other traditional risk factors. Thus, the evaluation of inflammatory biomarkers, such as C-reactive protein, and its lowering, could be critical [61]. As mentioned above, the present study demonstrated a significantly decreased hsCRP level compared to baseline values only in the n-3 PUFAs group (Table 2), indicating the anti-inflammatory effect of n-3 PUFAs. Thus, we confirmed the results of our earlier study, in which young healthy people received n-3 PUFAs supplements in the form of eggs [50], whereas some studies did not find a significant impact of a moderate n-3 PUFA supplementation (fish oil daily intake of 1400 mg EPA+DHA for 18 weeks) on the CRP value in healthy subjects [62,63]. On the other hand, several human studies have shown inverse associations between EPA and DHA status and blood markers of inflammation, such as C-reactive protein [64,65,66]. In hypertensive and/or diabetic obese adults, daily supplementation with fish oil capsules containing 300 mg EPA and 200 mg DHA for two months has a favorable effect on lowering hsCRP levels [67]. Considering the undoubted association between systemic inflammation biomarkers and CV disease [68], it is indicated that n-3 PUFAs consumption reduces the risk for future CV events, not only in conditions of disease and inflammation but also in health. Thus, n-3 PUFAs could be recommended as adjuvant anti-inflammatory agents, but it is crucial to consider whether the duration or dosage used is sufficient to achieve optimum treatment effectiveness.

Furthermore, numerous clinical and epidemiological studies suggest a positive role for n-3 PUFAs in CV disease outcomes [69,70,71,72]. Several mechanisms underlying the anti-inflammatory effect of n-3 PUFAs include the altered composition of cell membrane fatty acid phospholipids, impairment of lipid rafts, and prevention of activation of the pro-inflammatory transcription factor [73]. Furthermore, n-3 PUFAs serve as a substrate for synthesizing specialized pro-resolving mediators, such as resolvins, which play a crucial role in the resolution of inflammation [74,75,76]. Among the resolvins, RvD1 and RvE1 are distinct in their ability to resolve inflammation by actively shutting down the inflammatory response [74], without causing immune suppression [77], and, thus, represent promising therapeutic compounds. Our study results demonstrated that the serum concentrations of both RvE1 and RvD1 significantly increased following the dietary protocols in the n-3 PUFAs group, while the dietary intake of regular chicken meat did not affect their levels (Figure 8). Among others, RvE1 and Rvd1 underline the majority of the favorable effects assigned to their precursors [78]. Therefore, understanding the processes involved in controlling and resolving inflammation provides insight into the prevention and treatment of inflammatory diseases of various organs.

Although the beneficial effects of n-3 PUFAs are widely recognized, increasing evidence suggests that these fatty acids initially have a pro-oxidative effect that stimulates the antioxidant pathway. This response could be therapeutically valuable for conditions related to inflammation and oxidative stress [79,80]. To evaluate the potential impact of n-3 PUFA-enriched chicken meat on antioxidant status, we measured the serum levels of TBARS and FRAP formation, using widely accepted and well-described methods [39,42]. The TBARS method was used to evaluate the extent of lipoperoxidation, whereas the FRAP assay was used to measure the antioxidant potential. We found no significant difference in TBARS levels after consuming both regular and n-3 PUFA-enriched chicken meat (Figure 2), whereas n-3 PUFA-enriched chicken meat consumption significantly increased FRAP levels (Figure 3). Taking into account that dietary supplementation with n-3 PUFAs reduces oxidative stress-related mitochondrial impairment and cell apoptosis by increasing the activity of endogenous antioxidant enzymes [32], we further evaluated the serum GPx and SOD activities. Antioxidant enzymes, such as glutathione peroxidase (GPx) and superoxide dismutase (SOD), are a primary defense system against oxidative stress [81]. Though these scavenger enzymes are initially inactivated when counteracting ROS, the presence of these species induces their expression [82]. After a three-week dietary intake of n-3 PUFA-enriched chicken meat, GPx and SOD activities significantly increased compared to baseline (Table 3), while they remained unaltered following regular chicken meat consumption. Furthermore, the consumption of n-3 PUFA-enriched chicken meat significantly reduced the intracellular formation of hydrogen peroxide and peroxynitrite (Figure 4 and Figure 5) and had no significant effect on superoxide formation (Figure 6 and Figure 7) in peripheral blood mononuclear cells. Enhanced GPx activity could explain the reduced level of hydrogen peroxide and peroxynitrite, while enhanced SOD activity could be the reason for the unchanged level of superoxide production. This indicates a promising effect of the n-3 PUFA-enriched diet in mitigating intracellular oxidative stress. Alongside their anti-inflammatory and pro-resolution properties, n-3 PUFAs have been found to reduce pro-oxidant activity by enhancing the expression of protective antioxidant proteins. These findings suggest that dietary intervention may modulate cellular oxidative stress dynamics, thus potentially offering a novel avenue for managing oxidative stress-related conditions or pathologies. Meat and meat products are excellent food sources for delivering bioactive compounds, particularly n-3 PUFAs, by increasing the nutritional quality and adequacy of the diet [79,83]. The present study results support the beneficial effect of n-3 PUFA supplementation, specifically in the form of enriched chicken meat as a functional food.

Our study has some limitations. The short time of the intervention is a limitation of this study. Generally, a minimum of one month is needed for an intervention duration, while our study utilized a three-week dietary protocol. However, despite only 3 weeks of dietary protocol, our results demonstrated a decrease in cellular oxidative stress and an increase in cellular and plasma antioxidative capacities with the ingestion of n-3 PUFA-enriched meat. Furthermore, only young healthy people were included in this study; therefore, there was no inflammation or impairments that required mitigation in such a sense. On the other hand, this could also be a strength of this study, since the most consistent evidence for the favorable effect of n-3 PUFAs is in CV patients, and the effect in the state of general health is less investigated.

## 5. Conclusions

**Figure 9:**
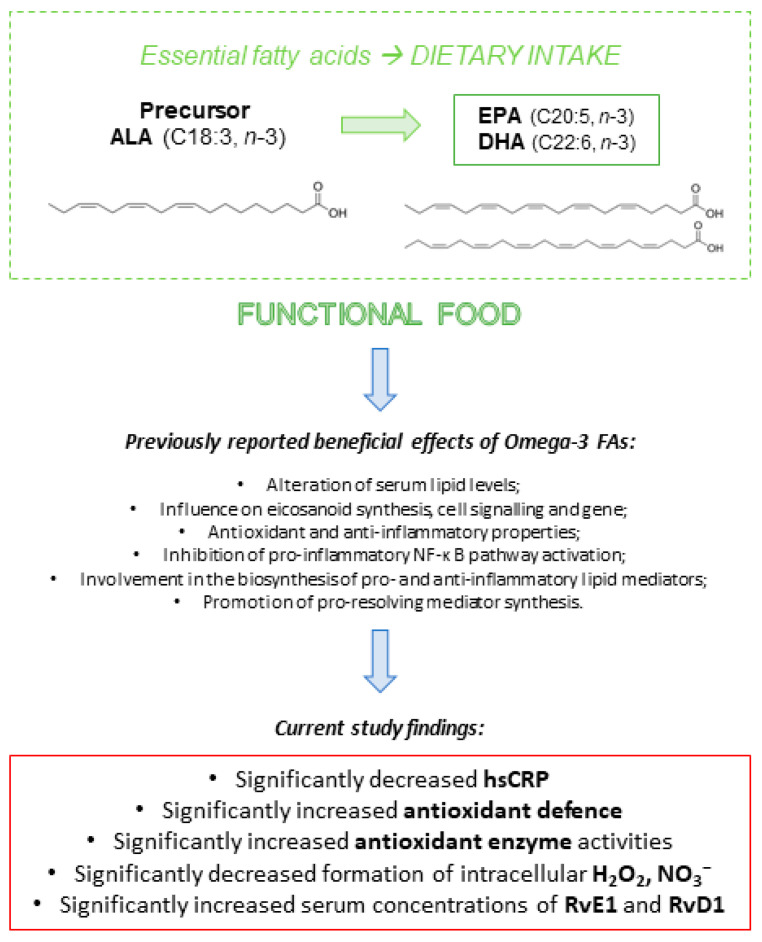
Scheme showing the most important mechanisms by which n-3 PUFAs could decrease inflammation and reduce the body’s oxidative stress, thus exerting a beneficial effect on human health.

Consuming chicken meat enriched with n-3 PUFAs may influence the physiological processes related to oxidative balance in young and healthy individuals. Specifically, a daily intake of approximately 1500 mg of n-3 PUFAs for three weeks is linked to reduced production of intracellular H_2_O_2_ and NO_3_^−^. Additionally, this intake has been associated with increased serum FRAP levels and enhanced activity of the GPx and SOD enzymes, as well as elevated levels of RvE1 and RvD1. Our findings indicate that n-3 PUFAs, when consumed through enriched chicken meat, can serve as effective antioxidants. This can help in preventing or reducing oxidative stress in general health conditions. Based on our current study and previous research [4,32], the possible mechanisms through which n-3 PUFAs may decrease inflammation and lower the body’s oxidative stress are illustrated in Figure 9.

## Figures and Tables

**Table 1:** Fatty acid profile of chicken meat (breast and tight muscle) of regular (eaten by controls) and n-3 PUFA-enriched meat (eaten by n-3 PUFAs group).

	Fatty Acid	Regular Chicken Breast (mg/100 g Meat)	n-3 PUFA-Enriched Chicken Breast (mg/100 g Meat)
Chicken breast	∑SFA	626.27 (28.45)	602.38 (50.64)
	∑MUFA	627.54 (47.69)	694.13 (76.42) *
	∑n-6 PUFA	824.47 (50.59)	451.98 (36.58) *
	ALA	17.74 (3.33)	177.63 (21.75) *
	Eicosatrienoic acid	0.00 (0.0)	9.01 (3.77) *
	EPA	0.00 (0.0)	25.07 (6.35) *
	DHA	0.00 (0.0)	33.75 (13.76) *
	∑n-3 PUFA	17.74 (3.33)	245.46 (13.95) *
	∑n-6 PUFA/∑n-3 PUFA	46.48 (7.84)	1.84 (0.17) *
Chicken thigh muscle	∑SFA	1654.46 (120.95)	1684.10 (68.53) *
	∑MUFA	2197.44 (168.53)	2281.36 (174.38) *
	∑n-6 PUFA	2217.87 (241.25)	1200.99 (88.64) *
	ALA	46.27 (10.56)	501.54 (36.94) *
	Eicosatrienoic acid	0.00 (0.0)	14.98 (8.56) *
	EPA	0.00 (0.0)	45.57 (16.85) *
	DHA	0.00 (0.0)	55.81 (28.91) *
	∑n-3 PUFA	46.27 (10.56)	617.90 (62.25) *
	∑n-6 PUFA/∑n-3 PUFA	47.93 (13.49)	1.94 (0.15) *

Data are presented as mean and standard deviation (SD). ∑SFA—saturated fatty acids (C14:0, C15:0, C16:0, C17:0, C18:0, C20:0, C21:0, C23:0); ∑MUFA—monounsaturated fatty acids (C14:1, C16:1,C18:1n9t, C18:1n9c, C20:1n9, C22:1n9); ∑n-6 PUFA—polyunsaturated fatty acids (C18:2n6c, C18:3n6, C20:3n6, C20:4n6, C22:2n6); ALA—alpha linolenic acid (C18:3n3); Eicosatrienoic acid (C20:3 n-3); EPA—eicosapentaenoic acid (C20:5n3); DHA—docosahexaenoic acid (C22:6n3); ∑n-3 PUFA—polyunsaturated fatty acids (C18:3n3, C20:3n3, C20:5n3, C22:6n3). * *p* < 0.05 One-Way ANOVA Regular vs. n-3 PUFA-enriched chicken meat.

**Table 2:** General characteristics and biochemical parameters of the examined groups.

Parameter	Control Group			n-3 PUFAs Group			*p* ^‡^
N (W/M)	20 (12/8)			19 (8/11)			0.527
Age (years)	23 (3.0)			23 (3.0)			0.476
	Before	After	*p* ^†^	Before	After	*p* ^†^	
BMI (kg/m^2^)	24.8 (4.9)	24.6 (5.0)	0.978	23.9 (3.1)	24.0 (3.1)	0.871	0.366
WHR	0.81 (0.05)	0.82 (0.05)	0.813	0.82 (0.05)	0.82 (0.05)	0.854	0.975
erythrocytes (×10^12^/L)	4.6 (0.5)	4.7 (0.5)	0.551	5.0 (0.4)	5.0 (0.3)	0.429	0.958
hemoglobin (g/L)	132.6 (13.4)	135.2 (14.9)	0.658	142.6 (15.1)	141.9 (10.7)	0.429	0.994
leukocytes (×10^9^/L)	6.9 (1.7)	6.4 (1.8)	0.435	6.4 (0.8)	6.1 (1.0)	0.345	0.007*
thrombocytes (×10^9^/L)	258.0 (66.4)	260.4 (66.5)	0.928	267.3 (60.7)	254.2 (55.4)	0.978	0.911
urea (mmol/L)	4.8 (1.4)	5.6 (1.1)	0.096	5.2 (1.4)	5.8 (1.5)	0.093	0.47
creatinine (μmol/L)	71.4 (13.0)	73.6 (11.8)	0.735	73.6 (8.0)	76.4 (8.0)	0.091	0.26
sodium (mmol/L)	138.7 (1.3)	138.7 (1.4)	0.885	138.7 (1.7)	139.2 (1.8)	0.422	0.353
potassium (mmol/L)	4.3 (0.3)	4.2 (0.2)	0.115	4.5 (0.4)	4.5 (0.3)	0.283	0.083
calcium (mmol/L)	2.8 (0.1)	2.4 (0.1)	0.093	2.5 (0.1)	2.4 (0.1)	0.06	0.232
iron (µmol/L)	13.4 (6.1)	14.7 (7.5)	0.735	18.5 (7.4)	19.2 (5.0)	0.434	0.216
transferrin (g/L)	2.8 (0.4)	2.8 (0.5)	0.211	2.8 (0.4)	2.8 (0.4)	0.71	0.356
glucose (mmol/L)	4.8 (0.5)	4.5 (0.5)	0.09	5.0 (0.5)	5.0 (1.1)	0.724	0.279
hsCRP (mg/L)	2.1 (3.7)	1.2 (1.2)	0.755	1.5 (2.6)	0.8 (0.4)	0.364	0.002 *
cholesterol (mmol/L)	4.0 (0.6)	4.1 (0.7)	0.921	4.3 (0.8)	4.2 (0.9)	0.654	0.963
triglycerides (mmol/L)	0.9 (0.3)	0.8 (0.5)	0.184	0.9 (0.5)	0.7 (0.3)	0.731	0.602
HDL cholesterol (mmol/L)	1.5 (0.3)	1.5 (0.3)	0.09	1.5 (0.3)	1.5 (0.3)	0.505	0.096
LDL cholesterol (mmol/L)	2.2 (0.4)	2.4 (0.6)	0.121	2.5 (0.6)	2.6 (0.6)	0.105	0.987
AST (U/L)	23.3 (4.1)	28.4 (16.9)	0.282	26.4 (7.4)	24.1 (7.0)	0.06	0.23
ALT (U/L)	20.8 (5.7)	26.8 (16.0)	0.227	25.7 (17.3)	21.5 (11.7)	0.323	0.119
GGT (U/L)	13.9 (2.9)	15.5 (3.8)	0.06	17.7 (11.4)	21.0 (17.8)	0.118	0.451

Data are presented as mean and standard deviation (SD). N—number of participants; W—women; M—men; BMI—body mass index; hsCRP—high-sensitivity C-reactive protein; HDL—high-density lipoprotein; LDL—low-density lipoprotein; AST—aspartate aminotransferase; ALT—alanine aminotransferase; GGT—gamma-glutamyl transferase. *p* ^†^ before vs. after within the group (Control or n-3 PUFAs)—paired *t*-test; *p* ^‡^ between groups with adjustments for baseline (analysis of covariance (ANCOVA)); * *p* < 0.05.

**Table 3:** Antioxidant enzyme (CAT, GPx, and SOD) activity in regular (control group) or n-3 PUFA-enriched chicken meat dietary intake (n-3 PUFAs group).

Parameter (U/mg Protein)	Control Group		n-3 PUFAs Group	
	Before	After	Before	After
GPx	0.076 (0.02)	0.084 (0.01)	0.079 (0.02)	0.099 (0.03) *
SOD	14.08 (1.03)	14.39 (1.34)	14.19 (1.1)	14.87 (0.74) *

Results are expressed as mean and standard deviation (SD). CAT—catalase; SOD—superoxide dismutase; GPx—glutathione peroxidase. * *p* < 0.05 before vs. after within the n-3 PUFAs group; paired *t*-test.

## Data Availability

The original contributions presented in this study are included in the article; further inquiries can be directed to the corresponding author.

## Supplementary Materials

The following supporting information can be downloaded at: https://www.mdpi.com/article/10.3390/antiox14020204/s1, Table S1: CONSORT checklist.
